# Intelligent Tracking of Mechanically Thrown Objects by Industrial Catching Robot for Automated In-Plant Logistics 4.0

**DOI:** 10.3390/s22062113

**Published:** 2022-03-09

**Authors:** Nauman Qadeer, Jamal Hussain Shah, Muhammad Sharif, Muhammad Attique Khan, Ghulam Muhammad, Yu-Dong Zhang

**Affiliations:** 1 Department of Computer Science, Wah Campus, COMSATS University Islamabad, Wah Cantonment 47040, Pakistan; nauman.qadeer@fuuast.edu.pk (N.Q.); sharif@ciitwah.edu.pk (M.S.); 2 Department of Computer Science, Federal Urdu University of Arts, Science & Technology, Islamabad 45570, Pakistan; 3 Department of Computer Science, HITEC University Taxila, Taxila 47080, Pakistan; attique.khan@hitecuni.edu.pk; 4 Department of Computer Engineering, College of Computer and Information Sciences, King Saud University, Riyadh 11543, Saudi Arabia; 5 Department of Informatics, University of Leicester, Leicester LE1 7RH, UK; yudong.zhang@leicester.ac.uk

**Keywords:** real-time trajectory prediction, mechanically thrown objects, internal logistics, smart manufacturing systems, multi-camera simulation, many-to-many time series forecasting, encoder-decoder bidirectional LSTM deep neural networks

## Abstract

Industry 4.0 smart manufacturing systems are equipped with sensors, smart machines, and intelligent robots. The automated in-plant transportation of manufacturing parts through throwing and catching robots is an attempt to accelerate the transportation process and increase productivity by the optimized utilization of in-plant facilities. Such an approach requires intelligent tracking and prediction of the final 3D catching position of thrown objects, while observing their initial flight trajectory in real-time, by catching robot in order to grasp them accurately. Due to non-deterministic nature of such mechanically thrown objects’ flight, accurate prediction of their complete trajectory is only possible if we accurately observe initial trajectory as well as intelligently predict remaining trajectory. The thrown objects in industry can be of any shape but detecting and accurately predicting interception positions of any shape object is an extremely challenging problem that needs to be solved step by step. In this research work, we only considered spherical shape objects as their3D central position can be easily determined. Our work comprised of development of a 3D simulated environment which enabled us to throw object of any mass, diameter, or surface air friction properties in a controlled internal logistics environment. It also enabled us to throw object with any initial velocity and observe its trajectory by placing a simulated pinhole camera at any place within 3D vicinity of internal logistics. We also employed multi-view geometry among simulated cameras in order to observe trajectories more accurately. Hence, it provided us an ample opportunity of precise experimentation in order to create enormous dataset of thrown object trajectories to train an encoder-decoder bidirectional LSTM deep neural network. The trained neural network has given the best results for accurately predicting trajectory of thrown objects in real time.

## 1. Introduction

Smart manufacturing system is a modern form of production system which consists of industrial robots, numerically controlled machines, sensors, and standalone systems such as inspection machines. It uses semi-dependent workstations and material handling systems designed to efficiently manufacture more than one type of part ranging from low to medium volume [1]. The use of computer-controlled machines and robots in the production segment of manufacturing industries promises a variety of benefits ranging from high utilization to high productivity volume [2]. Replacing people with industrial robots is the long lasting demand of production systems. In order to cope up with this demand, industrial robots are going to be common in factories day by day, and a lot of work has been carried out in this dimension. However, still there is big horizon to explore in the domain of intelligent industrial robots. There is no single definition of intelligence, but the main features that characterize intellectual ability are judgment and adaptation of environment as well as on-the-spot solving of newly occurring problems. The work at hand is a step towards this dimension. Realizing the importance of manufacturing systems especially internal logistics (transportation of parts during manufacturing within the plant), it investigates a new approach for material transport within flexible manufacturing systems by a throw and catch technique implemented by intelligent industrial robots sensing through multiple cameras.

**Figure 1:**
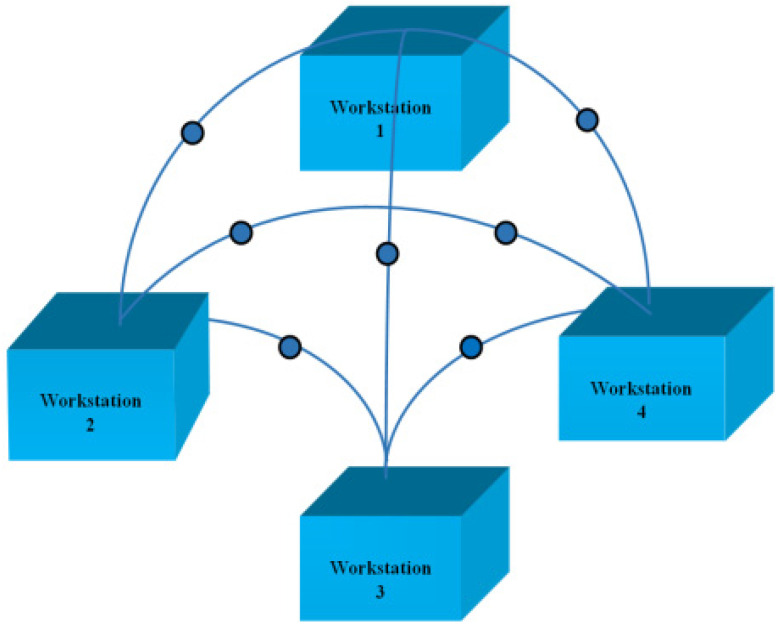
Automated in-plant logistics in industry 4.0 by intelligent throwing-catching robots at different workstations (using different altitude routes).

The aim of this research work is to explore the fastest way of transportation of parts as well as the optimum usage of the workspace of manufacturing plants, which can ultimately reduce manufacturing costs. Being the most direct connection between two places, the approach of throw and catch should be the fastest possibility for transportations. The basic principle of this transport approach is that a work piece in manufacturing plant is thrown by a robot to the subsequent workstation where it has to be caught by another robot. The catching is performed with a gripper so that the work piece can be handled variably in further production process steps. This approach is shown in Figure 1.

By applying throw-catch approach for transportation of objects in production systems, the fully automation can be achieved in flexible manufacturing systems which lead to the following advantages:

- Fast transportation
- Productivity increase
- Flexibility
- Optimized utilization of facilities
- Improved safety

The hurdle to implement this approach in industries so far is lack of intelligence in catching robot, as in industrial applications there are many kinds of unsymmetrical objects that can be thrown. Also, the exact position, angle and acceleration of thrown objects is not always known. Furthermore, air resistance and gravitational force are additional factors that influence the flight trajectory of such thrown objects. Due to these non-deterministic parameters, it is not possible to exactly determine the catching location of such thrown objects.

The flight trajectory of a mechanically thrown object can be observed by using optical sensors (i.e., cameras). However, there can be observational errors depending upon the number and positioning of the cameras. We can minimize this observational error if we have multi-camera view by appropriate number of cameras placed at optimum positions in internal logistics settings. Furthermore, enough experimentation of throws is required in order to gain a large dataset to train a supervised machine learning algorithm. In order to gain such dataset, we developed a 3D simulated environment in which a spherical object of any properties (such as mass, diameter, surface air friction, etc.) can be thrown with any initial parameters (initial position, acceleration, throwing angle, etc.) and its trajectory can be captured by a simple pinhole camera placed anywhere within the simulated 3D environment. We used a pinhole camera model for our simulation since perspective projection of most of the modern cameras can be described well by the pinhole camera model [3]. We also employed multi-view geometry among simulated cameras in order to observe trajectories more accurately. Hence it provided us an ample experimentation opportunity in order to create enormous dataset of thrown object trajectories to train any machine learning algorithm.

LSTM deep neural networks provided best results in forecasting time series data [4,5,6,7] such as the flight trajectory data in our research problem. However, this algorithm had never been applied in this particular problem of predicting mechanically thrown objects’ trajectories. The reason is that such algorithms are data hungry and they need an enormous training in order to have good learning (this is not always possible physically due to limitations of practical settings). However, our simulation made it possible to have enough experimentation of throwing objects with multiple variations as well as having sufficiently captured images within very short time period of thrown object’s flight. Moreover, during experimentation, the observed trajectory’s intercepting positions are more accurate by applying multi-view geometry.

Our trained encoder-decoder bidirectional LSTM deep neural network has given the best results for predicting trajectory of thrown objects in real time. During testing of our model, the actual intercepting positions of the trajectory are compared with predicted intercepting positions. The next section presents the research work carried out in both academia and industries in this particular problem. Then, in Section 3, our proposed methodology is explained in detail. Afterwards, Section 4 gives testing results to assess successfulness of our work. Finally, Section 5 concludes this research work and gives some future directions.

## 2. Related Work

The first attempt to apply throwing and catching robots for internal logistics was made in 2006 [8] as a collective attempt by department of Electrical Engineering of Reinhold Würth University Germany and the PMD technologies Germany. This approach has advantages of high flexibility and few resources requirement [9]. Since the acceptance of approach is still not very high for real applications today, certain challenges have to be solved to make it applicable. Such challenges, as summarized in [8], are as follows:

- Mechanically throwing or shooting of objects;
- Tracking of the catching device;
- Catching mechanically thrown objects;
- Detecting thrown object on its trajectory

Work already conducted in production systems with respect to each challenge is described below.

### 2.1. Mechanically Throwing or Shooting of Objects

Heinz Frank and his team developed a prototype [10] of a mechanical throwing device responsible for throwing circular objects in production systems. Through this device, the characteristics for the acceleration of the objects can be modified only by mechanical settings. With this device, circular objects with masses up to 70 g are thrown with speeds up to 10 m/s over distances up to 3 m. The spring of this throwing device can be compressed with a hydraulic jack and it can be released with a solenoid. Different accelerations of circular object can be set by variation in compression strength and that can be made by changing the distance between the bearing and solenoid.

In [11], Smith, C. and Christensen, H.I. have demonstrated a mechanical ball launcher which was used to throw soft juggling type ball with nearly 6 m/s velocity and they used it in their ball catching experiments where it was placed at the distance of approximately 4 m from the catching robot. Heinz Frank continued this work with cylindrical objects and, in [12], he along with his team introduced another throwing device that could throw cylindrical shaped objects over distances of about 3 m. In this throwing device, a pneumatic cylinder drives over an arm with leverage which can accelerate the object. The speed of throwing can be controlled with the pressure for the cylinder. It is able to throw cylinders with masses up to 100 g and of diameters up to 100 mm. Moreover, the cylinders thrown through this device maintain stable orientation and high targeted precision.

### 2.2. Tracking of the Catching Device

In [8], Objects to be transported are thrown in *x*-direction towards catching device. So, in order to catch objects, the capturing device only needs to move in two motion axes (*y*-axis and *z*-axis). 3D video camera is attached on capturing device. It can detect thrown object within its field of observation in intervals of 20 ms so with a flight time of 300 ms, 15 positions can be collected with this camera within the range of 3 m. It means, position is measured by camera after nearly 200 mm distance in *x*-axis (Δx). The accuracy in measurement of position can be judged by distance to the camera. As the distance of object from camera reduces, there is more precision in camera measured position of object. These factors are considered to track catching device. In [13], Cartesian robot is proposed as capturing device. This robot can move in *y* and *z*-axis. The vision is made is through a single camera mounted at top of robot. As the ball comes nearer to robot, its more precise capturing position can be predicted. Hence, the robot is moved accordingly to capture the object.

### 2.3. Catching Mechanically Thrown Objects

To catch fast flying objects in production systems, grippers are required which should have the closing time of less than 10 ms [13]. These grippers can have one or many jaws so that objects of many shapes can be captured. In [14], two types of grippers are proposed to capture flying objects in production systems. First type of grippers is that which use kinetic energy of the flying object to close the gripper. When the flying object enters into the jaw of gripper, it impacts a ram which pushes the lever and the lever closes the gripper without any reaction-delay. After that, object can be released by a linear drive in slow movement through the slope. The second type of grippers is mechanical in nature. With such grippers, the jaw is closed by a pre-stressed rotary spring. The closing movement is released by a ram. When flying object enters into jaw of gripper, the ram is pushed that pulls lever and rotary spring catches the flying object. This gripper is better for light weight objects. It is the kind of gripper that is used as a capturing device for a Cartesian robot in [13].

### 2.4. Detecting Mechanically Thrown Object on Its Trajectory

There are several challenges for tracking of thrown objects such as continuously changing background, aerodynamic stability and dynamic appearance throughout flight [15]. In order to meet these challenges, a lot of work had already been carried out. Most of the work is in sports domain such as soccer [16,17], cricket [18,19], basketball [7,20], tennis [21], and ping-pong playing robots [22,23,24,25]. Some work is also carried out for catching robots such as the work in [26] for a ball catching robot where a ball 8.5 cm in diameter was wrapped in retro-reflective foil and its flight trajectory was observed through stereo triangulation by two cameras mounted as the eyes of a catching humanoid robot. The reflective foil made ball more fluorescent, and hence it was easily detectable by humanoid robot. The EKF (extended Kalman filter) was used for prediction based upon the flight interception positions judged 0.16 to 0.2 s before catch. That humanoid robot caught 80% successful catches, whereas a successful catch was made whenever the prediction was within range of 15 mm error. However, the total numbers of test trajectories were not specified in that article. A similar work was also conducted in [27] where the article claim 98% successful catches by robot capable of catching tennis ball within error range of approximately 10 cm (i.e., 100 mm).

Except for ping-pong playing robots, all of the other above-mentioned work involves non-mechanical throws. The work of ping-pong playing robots such as [22,23,24,25] involves mechanical throw of ball as ping-pong ball is served mechanically by robotic arm. Mechanically thrown objects in production systems are unsymmetrical and not absolutely identical. So, their trajectories are influenced by different factors such as different conditions during the acceleration, the influence of the gravitation and aerodynamic resistance. Therefore, for catching such objects, the trajectory of objects must be observed online during the flight [8]. Some efforts have already employed in this regard. The work in [28] used 3D video range camera to observe flight trajectory. Such a camera employs PMD (photonic mixer device) principle which uses 3D optical detection to measure time-of-flight and it was developed by PMD Technologies. Such high-speed 3D camera was based on 64×48 pixel photonic mixer device (PMD) sensor array and enables 3D distance measurements to be made with 50 fps speed. Using PMD, the distance data is acquired using time-of-flight principle with invisible near-infra-red light.

There is very limited work that is specifically carried out for tracking mechanically thrown objects regarding robotic throw catch approach. Those works involve object throws through a mechanical launcher. For example, the work in [29,30,31] used single camera on the catching side and trajectory of thrown objects was predicted by combination of two methodologies for determination of 3D positions of a thrown object during its trajectory. The first methodology was applied in early phase of trajectory and used measurement of the initial parameters (angles, position and velocity) of the thrown object. The speed of the object was measured by six photoelectric sensors placed at 40 mm vertical distance on an aluminum frame. While calculating the velocity along the approaching axis, aero-dynamic resistance had also taken into account. Hence by simply applying model of flight kinematics, 3D positions of object are predicted at specific time intervals in the initial phase of flight. In the next phase of flight trajectory, back-projection of 2D image object position to 3D real world position was made. This back-projection methodology was not used in earlier phase of flight trajectory since good accuracy (in determination of 3D position of ball) cannot be achieved when the object is far from the camera.

The work in [32,33] used stereo vision to collect samples of mechanically thrown tennis ball trajectories. However, they enabled researchers to measure positions of ball in camera-related coordinate system with millimeters of accuracy (even outliers in some measurements). They used KNN regression approach for forecasting on 2D points of camera coordinates. In their experiments they achieved nearly 30 mm precision in predicting future position of ball. Work in [34] used a simple neural network containing only one hidden layer. This NN was trained using 15 simulated trajectory training sets whereas each set had 10 sample trajectories obtained in nearly 2.5 m long flight of tennis balls that was mechanically thrown. The training was made using MATLAB Neural Networks Toolbox. The mean error was nearly 24 to 26 mm between measured values and prediction results in simulated environment.

Other mechanically thrown object’s flight prediction experiments were made by [35,36,37,38] using stereo vision based observational data as input to a forecasting algorithm responsible for generating trajectory using deterministic motion model further governed by genetic programming algorithm. Their algorithm was tested through MSE (mean square error) in chosen frames 60 to 80 and in only 20 test trajectories. The average MSE in 20 trajectories was 5.4 mm. Although this average MSE was good, it was based upon just 20 testing trajectories. As well, the error was calculated within selected frames and does not reflect the error of whole flight trajectory. The work carried out regarding mechanically thrown object’s trajectory tracking is summarized in the next section.

### 2.5. Limitations in Existing Work of Mechanically Thrown Objects Tracking

The limitations in existing work of mechanically thrown objects tracking are summarized in Table 1.

## 3. Proposed Methodology

As described in previous section, the majority of existing work for mechanically thrown object tracking, had chosen a tennis ball as the thrown object for development and validation of proposed ideas in order to implement robotic throw catch transportation approach in industry for small sized objects. This is due to the well-known aerodynamic properties of this object as there is already a number of scientific literature available that explored the aerodynamic properties of the tennis ball; a review of such exploration is given in [39]. Another reason is that detecting and accurately predicting interception positions of any shape object, during its flight, is an extremely challenging problem that needs a step-by-step solution. So, following this tradition, this research work also used tennis ball as mechanically thrown object.

**Figure 2:**
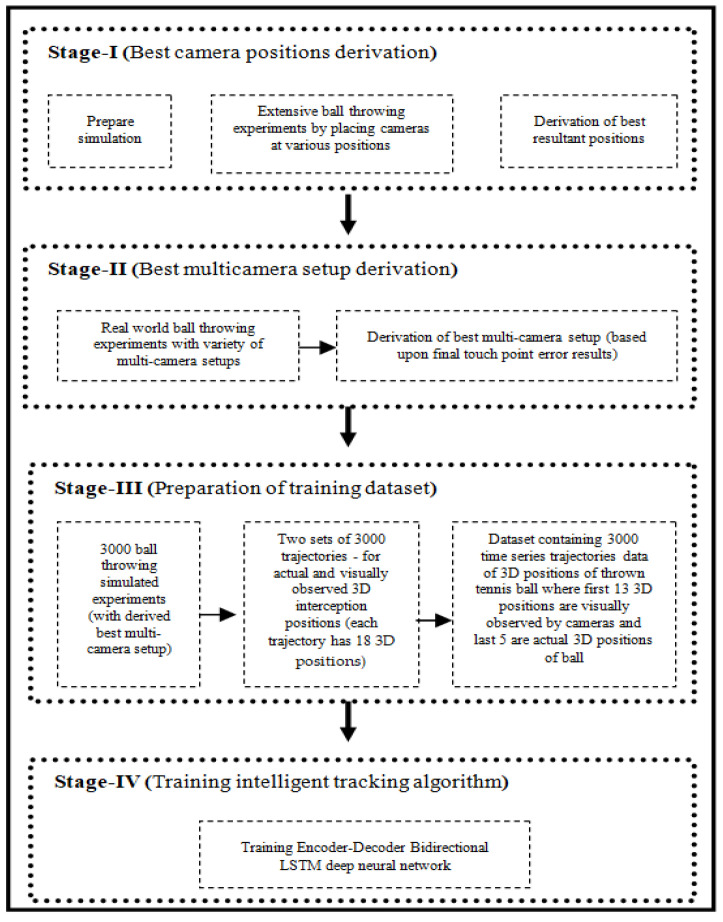
Proposed methodology.

This work consists of four sequential stages. In the first stage, a novel simulating environment is prepared that facilitates to throw any spherical object, in controlled logistics environment, and observe its trajectory by placing a simulated camera at any place within that 3D vicinity. It helped us to capture thrown object’s trajectory by placing camera at any place and finally the best camera positions are derived based upon the captured trajectory’s error. In the second stage, the best multi-camera setup is derived in a real world environment by placing cameras at optimum positions (derived in last stage) and also applying multi-view geometry among them. Then, in next stage, best derived multi-camera setup is used for thousands of experiments to prepare a comprehensive dataset of trajectories and in final stage an encoder-decoder Bi-LSTM deep neural network is trained. The overall proposed methodology is visually illustrated in Figure 2 and explained in detail in following subsections.

### 3.1. Simulation

The 3D vicinity of internal logistics environment is simulated in MATLAB as a cube with both positive and negative axis in all three dimensions. This simulation facilitates to throw spherical objects of any mass, diameter or surface air friction in a controlled environment. It also enabled us to throw object with any motion model and observe its trajectory by placing simulated pinhole camera anywhere within 3D vicinity of internal logistics in flexible manufacturing systems which is usually 3 to 5 m [8].

In fact, the perspective projection of most of the modern cameras can be described well by the pinhole camera model [5] and that is the reason for choosing pinhole camera model for implementation of virtual cameras in this simulation. It is to be noted that, in our simulation, virtual camera can capture trajectory with any frame rate but we used the frame rate of 60 fps in order to give it compatibility with real world experimental camera’s frame rate. Other properties of virtual camera (such as focal length, pixel pitch which is per pixel area on camera’s sensor, resolution of captured image etc.) are set as real world used camera (i.e., IDS UI-1220RE-M-GL) properties.

**Figure 3:**
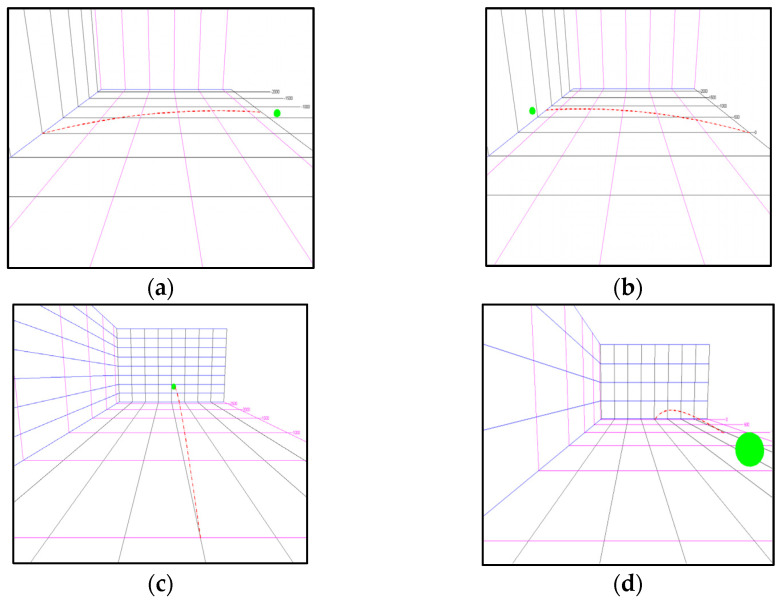
Last frame of trajectory (along with previous trajectory trace) when captured through four different simulated cameras. (**a**) One side camera view. (**b**) Other side camera view. (**c**) Throwing side camera view. (**d**) Catching side camera view.

All simulation used parameters are taken from our real-world experiments. In those experiments, the tennis ball is mechanically thrown towards *Y*-axis (towards catching robot) and the distance between throwing and final catching point is about 3 m. The initial position of ball is assumed at (0,0,0) in simulation. The ball’s radius is set as 32.2 mm (as in real world) and its coloris green in order to give the appearance of a real-world scene. Few visually captured trajectories, in our simulated throwing experiments are shown in Figure 3.

### 3.2. Experimental Setup and Simulation Testing

**Figure 4:**
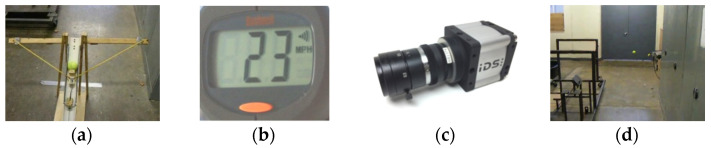
Real world experimental setup. (**a**) Ball throwing device. (**b**) Radar gun to measure launching speed. (**c**) Used camera (UI-1220RE-M-GL). (**d**) Trajectory captured through throw side camera.

We used a self-developed mechanical device for throwing tennis ball. This device throws ball by using kinetic energy that is produced by stretching a spring. The launching device is shown in Figure 4a. The launching speed of thrown ball is 23 m/h (i.e., 10.282 m/s) which is measured with the help of a radar gun shown in Figure 4b.

The used cameras are “UI-1220RE-M-GL” by IDS imaging development systems. This camera has sensor size of 1/3 inch, sensor area of 4.512 × 2.880 mm, focal length of 6 mm and per pixel area of 0.006 mm. It has the maximum capturing speed of 87 fps and its captured image has resolution of 752 × 480.

Before starting exhaustive simulated experiments for deriving best camera positions, the authenticity of our simulation was checked through a comparison experiment with exactly same parameters in real and simulated setups. There is always some distortion or deviation from ideal projection whenever a scene is captured by real world camera. It is due to several reasons but the prominent ones are lens quality and perspective projection effects such as an infinitely wide real-world plane is mapped onto the finite image area of lens. However, in spite of the deviational margin of results from ideal projection, the results from real and simulated experiments should be similar which authenticates the simulation.

The flight trajectory of thrown tennis ball was captured by placing a single camera at catching side in real world experiment. The exact measurements of camera and ball’s starting positions were (−262 mm, 2964 mm, 678 mm) and (0, 0, 0), respectively. The camera was oriented towards starting position of ball and exact measurements of orientation parameters are as follows: roll or tilt (orientation around *x*-axis) was measured as 23.40° degrees (clockwise), pitch or pan (orientation around *z*-axis) was measured as 25.13° degrees (anti-clockwise) and the yaw i.e., rotation around *y*-axis (the rotation of camera plane with respect to real world plane) was measured at 1.06° degrees (anti-clockwise). The same parameters were set in simulation experiment.

The tennis ball is mechanically thrown and the distance between throwing and final catching point is about 3 m. As mentioned earlier, the launching speed of thrown ball (measured through radar gun) was 23 m/h (i.e., 10.282 m/s). The launching angles of thrown ball can be named as alpha (left/right angle along *x*-axis) and beta (up/down angle along *z*-axis). The ball is thrown straight so ideally the alpha should be zero but in real world experiments the real value of alpha with precise measurement is 0.5 degrees. Similarly, the launching position makes the beta angle as 17 degrees. Knowing initial speed of ball and its throwing angles (alpha and beta), we can estimate the spherical coordinates (v, θ, φ) of initial velocity; where v is the initial speed of ball, θ is the azimuth angle (angle from the positive *x*-axis in *xy* plane) and φ is the inclination angle (angle from the positive *y*-axis in the *yz* plane). These spherical coordinates of initial velocity of ball (i.e., v, θ, φ) can be calculated as given in Equations (1)–(3).

Launching speed (v) = 23 miles/h

(1) i.e.,   V=23 × 0.44704 m/s=10.282 m/s

Azimuth angle (θ) = 90 − alpha = 90 − 0.5 = 89.5°(2)

Inclination angle (φ) = 90 − beta = 90 − 17 = 73°(3)

The cartesian coordinates of the initial velocity i.e., (*V_x_, V_y_, V_z_*) of thrown ball can be calculated using trignometric laws as given in Equations (4)–(6).

(4) $$V_{x}=V\cdot \sin\left(\varphi \right)\cdot \cos(\theta )=0.088\;m/s$$

(5) $$V_{y}=V\cdot \sin\left(\varphi \right)\cdot \sin(\theta )=9.830\;m/s$$

(6) $$V_{z}=V\cdot \cos\left(\varphi \right)=3.002\;m/s$$

In simulation, a tennis ball is launched with above initial parameters and its 3D trajectory is generated using standard ballistic motion model which also considers air density and spin of the ball under room temperature [40]. In our case, we used air density of 1.294 kg/m^3^ which is at 20 degree centigrade (similar to our lab temperature). The mass of ball, assumed in simulation, is the exact mass of our used tennis ball and that is 56 g. Similarly, the simulation used radius of tennis ball is 32.2 mm which is its real world radius. At starting point of ball, the time *t* = 0, so we can represent initial velocity by V(0) and if we ignore all forces (except gravity and drag) influencing the flight then the velocity (in any dimension) of ball at a particular time *t* (i.e., V(t)) and can be calculated by Equation (7) and the future velocities of ball can be derived by simple ballistic motion equation as shown by Equation (9).

(7) $$V\left(t\right)=\;V\left(0\right)+g\times t-\overset{t}{\underset{T=0}{\int }}\;k\times \;v^{2}\;dT\;$$

(8) where      g= [0, 0, 9.8]  

(9) i.e.,   V(t+Δt)= V(t)+(a(t)+g)×Δt

If the starting position of ball, i.e., at time *t* = 0, is assumed at (0, 0, 0) then every next position of ball (in any direction) can be calculated from current position (at time *t*) by using the following formula given in Equation (10).

(10) Pos(t+Δt)=Pos(t)+V(t)×Δt. 

**Figure 5:**
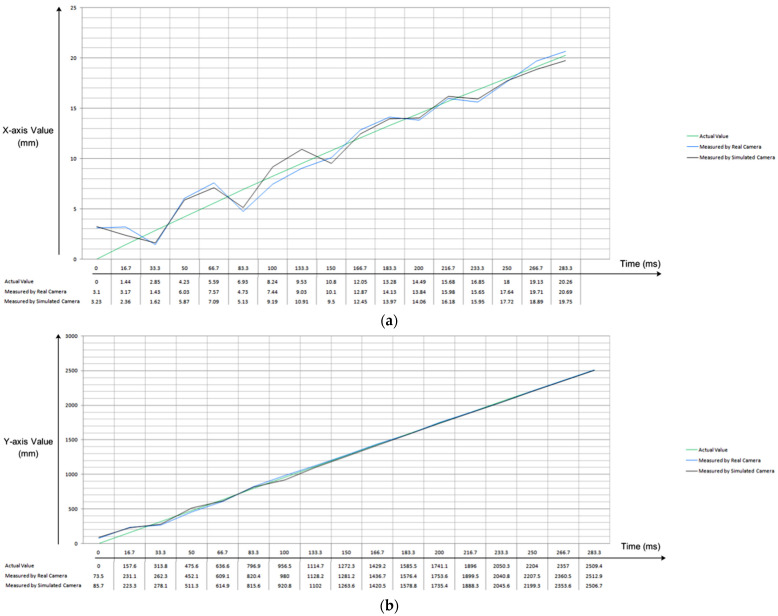

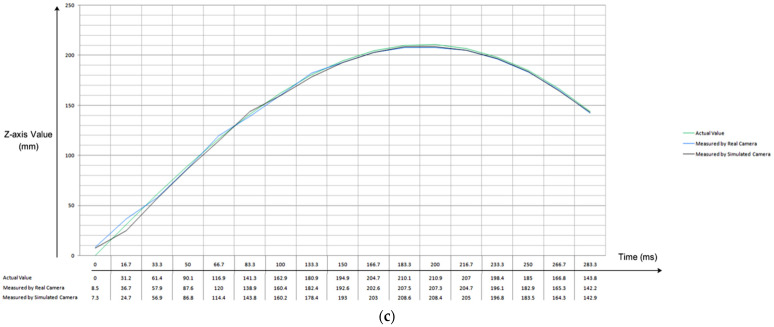
(**a**) Comparison of *X*-axis values (in actual and reconstructed trajectories by real & simulated camera). (**b**) Comparison of *Y*-axis values (in actual and reconstructed trajectories by real and simulated camera. (**c**) Comparison of *Z*-axis values (in actual and reconstructed trajectories by real and simulated camera).

When the trajectory is captured by real world camera, the total flight of moving ball is captured in 17 frames of movie which is captured at the speed of 60 fps. If we also consider the launching position of ball (i.e., 0,0,0) then the total 3D interception positions of flight trajectory are 18. The same parameters were used for simulation and hence the total flight time is 283.33 ms. The tennis ball is mechanically thrown and its 3D trajectory is captured through real camera whereas in simulation the trajectory is generated through ballistic motion model and it is captured by simulated camera working under pinhole camera model principles. In both real and simulated experiments, the tennis ball is thrown with the same initial velocities as mentioned by Equations (4)–(6). Both simulated and real cameras capture the full trajectory of thrown ball in 17 frames under the same capturing frame rate of 60 fps. The exact differences at each interception position (along *X*, *Y* and *Z* axes) are shown through graphs in Figure 5a–c, respectively.

The comparison of results (illustrated in Figure 5) has shown the close resemblance of real and virtual camera’s reconstructed trajectories as there are few millimeters differences between the three measured axis’ values for corresponding interception positions of flight trajectory. It is also observed that reconstructed trajectories (by both real and simulated cameras) had wider difference (from corresponding actual position values) in early stages of flight and the error reduces in later stages of flight; that is, as the thrown object gets closer to camera (which is mounted at catching side).

### 3.3. Best Multicamera Setup Derivation

After testing simulation, we can trust its trajectory results obtained by throwing same object (i.e., tennis ball) and employing simulated camera at any position and orientation within the simulated 3D vicinity of smart manufacturing plants. The process of deriving best multi-camera setup is divided into three phases. The first phase is to guess the areas where the best results can be found.

Assuming the starting position of ball as (0,0,0) and moving towards +ve *Y*-axis, it covers nearly the distance of 2.5 m (i.e., 2500 mm). So, seeing this, we set the initial 3D experimentation range for simulated camera placement. This initial range is −500 mm to 3500 mm for *Y*-axis, −500 to 1500 mm for *Z*-axis and −1500 mm to 1500 mm for *X*-axis. In order to guess best resultant areas, the simulated experimentation should be performed by placing camera at every 500 mm apart position within the selected 3D initial range. It is to be noted that these limits are chosen as the reasonable limits and can be changed (based upon initial experimentation results). For example, the *Z*-axis limit is chosen as −500 to 1500. But if the best results are obtained at *Z*-axis value of −500 then it shows a possibility to get better results beyond this value which ultimately extend the experimentation range so that *Y*-axis value of −1000 should also be considered (and even this process can be continued further). It gives the justification for choosing the above-mentioned axis limits in initial experimentation.

As the object is thrown straight and moves within a small *X*-axis value range (i.e., 0 to 25.3 mm), so the trajectories captured by placing the camera at particular (*X*, *Y*, *Z*) position and (−*X*, *Y*, *Z*) position are nearly symmetrical which implies that results obtained at these positions have minute difference. As only guess has to be made within this first phase that which area is better to do refined experimentation, so such minute difference can be neglected for initial experimentation and only positive X-values can be considered. The initial experimentation results have shown that minimum error was obtained at throwing and catching side cameras. So, further experimentation is carried outby placing cameras at 250 mm apart distances from initially guessed area. The results of these further experimentation eventually give us best resultant area which require refined experimentation to find out best capturing camera positions.

**Figure 6:**
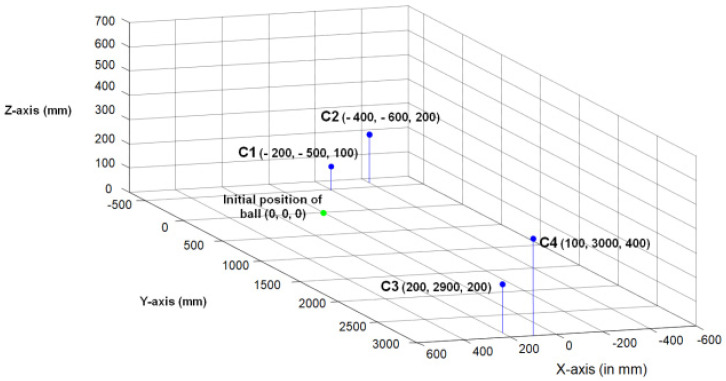
Identified best capturing cameras positions.

As we have to carry out refined experiments in the initially guessed area so we also include earlier neglected negative *x*-axis area also and now within this area, we performed refined experiments by placing camera at each 100 mm apart camera position. Finally, we got four best capturing cameras positions based upon the minimum trajectory error between actual and observed trajectory. We named those four best camera positions as C1, C2, C3 and C4 and they are precisely shown in Figure 6.

It should be noted that the minimum trajectory error is observed when the thrown object trajectory is captured from throwing and catching side cameras at reasonable distances. This is due to the fact that the size of the thrown ball changes significantly in videos when captured from those positions, and this significant change helps in proper identification of distance of ball from camera. Furthermore, reconstructed *X*-coordinate and *Z*-coordinates of ball are derived from the distance between camera and ball, so significant change in size of ball are required for proper reconstruction of all three coordinates of the real position of the ball.

Finally, in the last phase, different combinations of these best positioned cameras are employed in real world settings in order to derive best multi-camera setups in terms of maximum accuracy of final touch point 3D position error. It is the last position of ball which is measured on DST (dispersive signal technology) touch screen placed on catching side. Two-camera combinations are made in a way that they must contain both throw and catch side cameras in order to get more accurate results. Since it is necessary to include throw and catch side cameras so there are only four possible combinations of these cameras for two cameras setups. Similarly, there are four possible combinations to make three cameras setup. All of these two and three camera setups are shown in Table 2 along with their obtained experimental results which are given as average value of 50 ball throwing experiments.

The results shown in last row, by three cameras setup (i.e., C1 + C3 + C4), are the best ones. So, this setup is considered as best derived multicamera setup which is used in simulation for preparing training dataset of throws.

### 3.4. Preparing Throws Datasets and Training Intelligent Tracking Models

**Figure 7:**
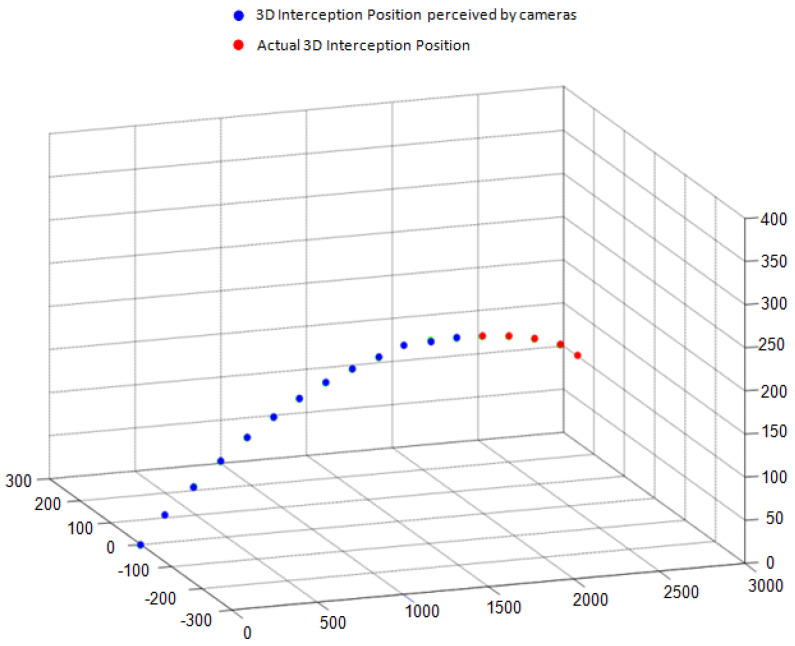
Sample dataset throw.

The best derived three cameras setup is employed in simulated environment and 3000 throwing experiments were performed (with very minute variation in throwing parameters). For each throwing experiment, two time series are recorded. The first one consists of actual 3D interception positions of thrown ball and second consists of 3D interception positions of balls as perceived by observing cameras. There are 17 moving 3D interception positions of ball in its trajectory when cameras captured 283 ms of flight trajectory of ball at capturing speed of 60 frames per second. So, there are total 18 captured video frames (including first frame when ball is placed at starting position (i.e., 0,0,0). We need to prepare a training dataset of throws where, in each dataset throw, first few 3D interception positions are cameras observed interception positions and last ones are actual 3D interception positions. So, considering this point, we used first 13 interception positions as perceived by cameras (it captured approximately first 200 ms of flight trajectory) and last five interception positions are actual 3D positions of ball (covering around 83 ms of last part of flight trajectory). We need to train an intelligent tracking model that gets input as camera perceived 3D interception positions of first 200 ms of thrown ball flight trajectory and predict next 83 ms of flight trajectory consisting of last five actual interception positions of the thrown ball. It provides enough time to instruct motor of modern catching robot to place catching gripper at predicted final 3D interception position. A sample throw of training dataset is shown in Figure 7.

The main contribution of this work is to propose an enhanced intelligent tracking algorithm that can predict remaining 3D interception positions of thrown object by seeing its initial flight trajectory. It is a pure time series prediction problem and LSTM deep neural networks work best for such problems. In particular, this is a many-to-many time series prediction problem where both input and output vectors consists of multiple time-step data values and each value further consists of three values (*X*, *Y*, and *Z* axis coordinate). The encoder-decoder LSTM is usually used for such many-to-many time series prediction problem. We have particularly used encoder-decoder bidirectional LSTM deep neural networks for this problem and they proved to be so much accurate as depicted by prediction results. The bidirectional LSTM preserves timeseries information both from forward and backward sequential contexts.

**Figure 8:**
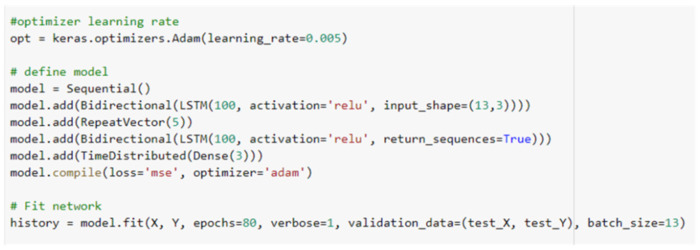
Coding (carried out in Keras) for training one of our encoder-decoder BiLSTM.

The encoder bidirectional LSTM creates an internal representation of the input sequence whereas the decoder bidirectional LSTM interpret this internal representation and responsible for predicting corresponding outputting sequence. The training is performed on 90% of throws dataset and model is validated on remaining 10% of dataset. The initial learning rate is set as 0.005. The model learned best with ‘Adam’ optimizer. We trained model (in Keras) using different number of epochs and neurons. For example, one of the training codes which we coded in Keras (Python deep learning API) is shown in Figure 8.

**Figure 9:**
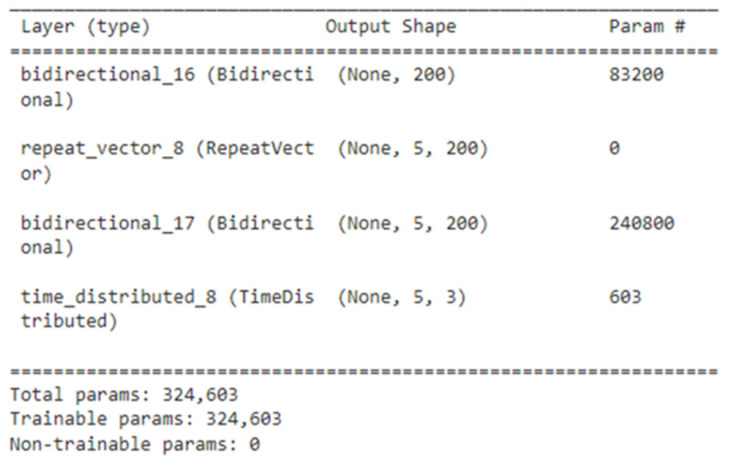
Summary of trained model (whose coding given in Figure 8).

This code used the input shape as (13.3), it is due to the fact that each of our training dataset throw has 18 three dimensional points and first 13 points of observed flight trajectory are given as input to encoder BiLSTM which consists of 100 neurons as input to each of its LSTM (i.e., forward and backward LSTMs). This encoder BiLSTM returns output as five points (i.e., the remaining five points of flight trajectory) that are received by RepeatVector which feeds them repeatedly as input to decoder BiLSTM which further has 100 neurons as input to both of its forward and backward LSTMs. The code also depicts that the output is of five time steps distributed where each time step value has three features (i.e., *X*, *Y*, and *Z* coordinates). The decoder BiLSTM in fact uses the value from RepeatVector, the hidden state from previous output and the current input. The return sequence of decoder BiLSTM is set as “true” since the output is in the form of time steps. The RepeatVector is used for only repeating the encoder LSTM output and it has no parameter to train. For example, see the summary of above-mentioned model in Figure 9.

The Figure 9 shows that RepeatVector has no parameter to train and it can also be seen, from summary, that each of the encoder and decoder bidirectional LSTM (BiLSTM) has 200 input neurons which is due to the fact that BiLSTM has two LSTMs and we have given 100 neurons as input to each LSTM.

**Figure 10:**
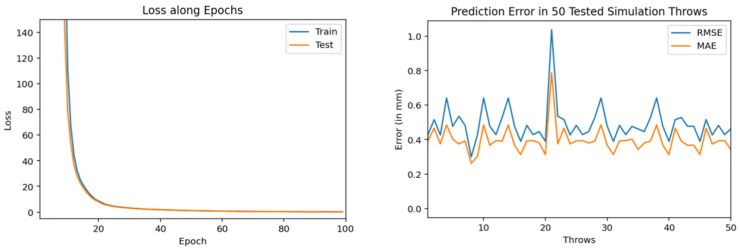
Training and testing results by encoder-decoder bidirectional LSTM deep NN (trained through 200 throws with 100 epochs and 100 neurons).

The experimentation of training and testing such models is carried out at different volumes of training dataset throws. Also, we trained and tested multiple encoder-decoder BiLSTM deep neural networks (with different number of neurons and epochs) in order to check their accuracy. We gradually increased the volume of training dataset which consists of simulation throws. Initially a model is trained using only 200 training throws dataset. It used 100 epochs (during training) and 100 neurons as input. In Figure 10, the loss along epochs is shown during its training. The prediction results, in root mean square error (RMSE) and mean absolute error (MAE), in 50 tested throws are also shown in this figure.

**Figure 11:**
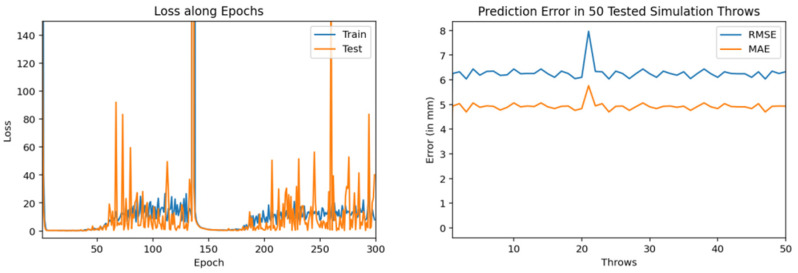
Training and testing results by encoder-decoder bidirectional LSTM deep NN (trained through 1000 throws with 300 epochs and 200 neurons).

**Figure 12:**
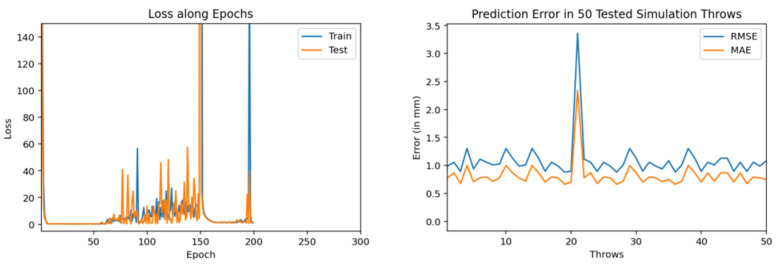
Training and testing results by encoder-decoder bidirectional LSTM deep NN. (trained through 1000 throws with 200 epochs and 100 neurons).

**Figure 13:**
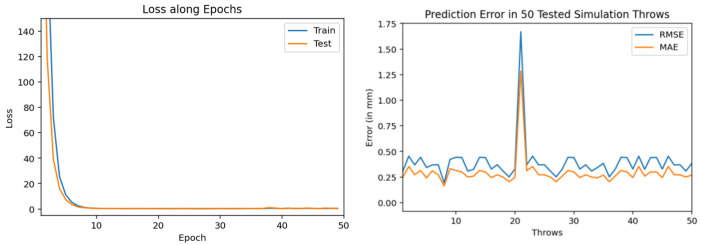
Training and testing results by encoder-decoder bidirectional LSTM deep NN (trained through 1000 throws with 50 epochs and 100 neurons).

Then, we trained similar models using 1000 throws dataset. As mentioned earlier, for every model training we used 90% dataset for training and 10% for validation. Figure 11, Figure 12 and Figure 13 show the training and testing (on 50 tested throws) performance results when encoder-decoder Bidirectional LSTM models are trained on 1000 throws datasets with varying number of epochs and neurons. The comparative analysis of all three of these models shows that over fitting can occur when we cross certain epoch limit and, in this particular case, we got the best results with 50 epochs.

**Figure 14:**
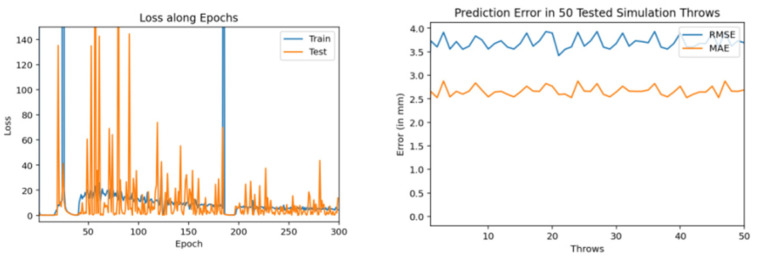
Training and testing results by encoder-decoder bidirectional LSTM deep NN (trained through 3000 throws with 300 epochs and 200 neurons).

**Figure 15:**
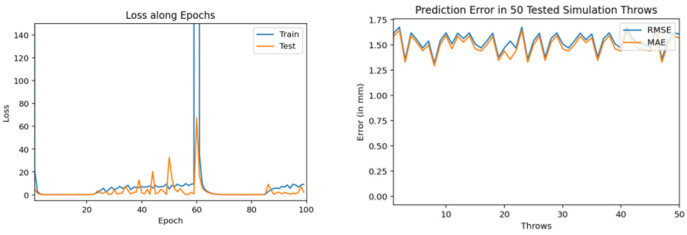
Training and testing results by encoder-decoder bidirectional LSTM deep NN (trained through 3000 throws with 100 epochs and 100 neurons).

**Figure 16:**
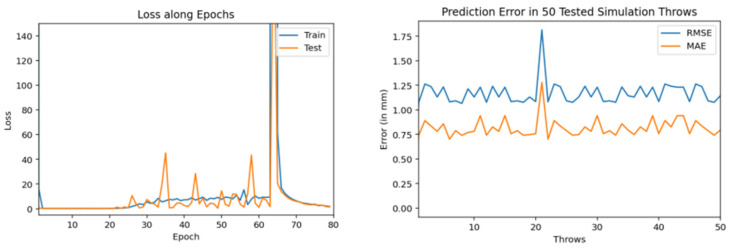
Training and testing results by encoder-decoder bidirectional LSTM deep NN (trained through 3000 throws with 80 epochs and 100 neurons).

Finally, we trained the complete dataset of 3000 throws. Figure 14, Figure 15 and Figure 16 show the training and testing (on 50 tested throws) performance results when encoder-decoder Bidirectional LSTM models are trained on complete dataset of 3000 throws with varying number of epochs and neurons. The comparative analysis of testing graphs of all of these three models shows that we get best results when model is trained using 80 epochs and with an input of 100 neurons to each LSTM in encoder-decoder bidirectional LSTM. This model is the best intelligent model (with maximum prediction accuracy) in our case. In next section, the testing results of this model are presented with a greater number of tested throws.

## 4. Results and Discussion

**Figure 17:**
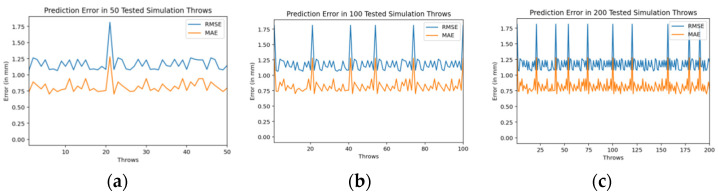
Prediction error results by applying proposed model for different datasets of test throws.

The encoder-decoder BiLSTM deep neural network trained on complete dataset of 3000 throws, with 100 neurons and trained through 80 epochs has presented best results and hence considered as our proposed intelligent tracking model for automated industrial catching robots responsible for in-plant logistics through throwing an catching manufacturing parts. Hence, we further test this trained intelligent tracking model on different throws datasets. Figure 17a–c shows prediction error graphs for three test datasets of 50, 100, and 200 throws, respectively.

**Figure 18:**
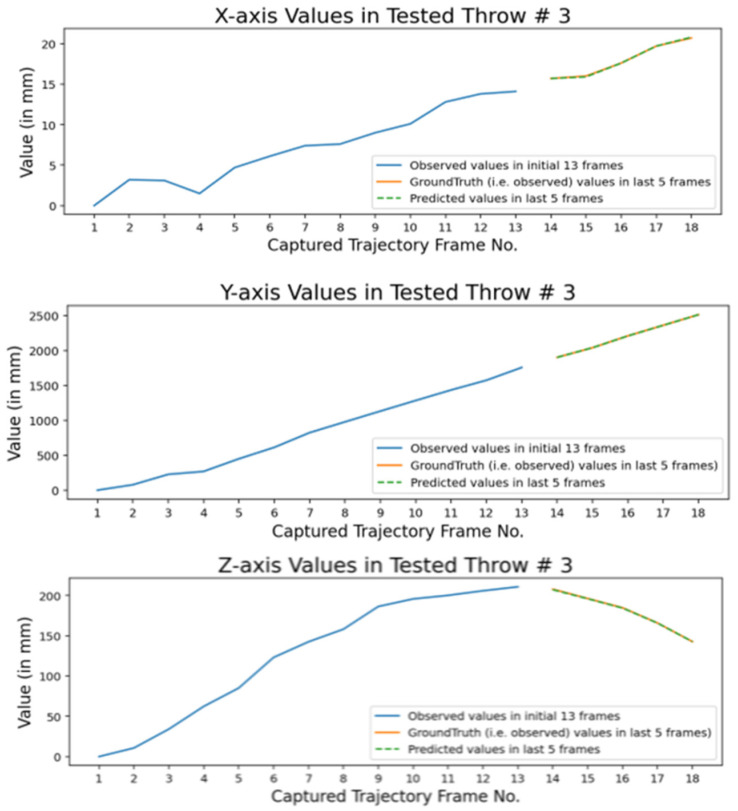
Comparison among predicted and ground truth values in a tested simulated throw.

These graphs show the accuracy of predicted values through our intelligent model. The obtained results are within the range of 2 mm error. In Figure 18, the graphs are shown, against each axis value, for 3D interception positions of a tested throw.

The first 13 are cameras observed values. The last five are actual (i.e., ground truth) values and predicted values (obtained through our trained intelligent model). The good overlap between predicted values and ground truth again represents the accuracy of our intelligent tracking model.

## 5. Conclusions and Future Work

The automated in-plant logistics for smart manufacturing systems of Industry 4.0 can be implemented through throwing and catching robots for fast transportation of small sized materials, containers, or packages. However, it needs to enhance the intelligence of existing catching robots by more accurate tracking of mechanically thrown objects. This work includes the development of a 3D simulated environment which enabled one to throw object of any mass, diameter, or surface air friction in a controlled internal logistics environment. It also enabled us to throw objects with any initial velocity and observe their trajectory by placing simulated pinhole camera at any place within a 3D vicinity of the internal logistics space. Moreover, the multi-view geometry can also be employed among simulated cameras in order to observe trajectories more accurately. The simulation further enabled us to derive the best multicamera setup and, after that, a dataset of 3000 throws was prepared using that setup in simulated environment. An intelligent tracking model is proposed using encoder-decoder bidirectional LSTM approach. The model predicts final part of thrown object flight trajectory by observing its initial flight through cameras and in real-time. This model is trained using prepared dataset and its prediction results are compared with ground truth values in simulated test throws. The trained neural network has given best results for accurately predicting the final part of mechanically thrown object trajectory in real time.

This research work only considered tracking of spherical shaped mechanically thrown objects as their 3D central position can be easily determined in video frames. In future, we have planned to extend this work for other regular shaped objects in order to implement it practically in industry for automated in-plant fast transportation of small sized materials such as containers, food, or pharmaceutical products.

## Figures and Tables

**Table 1:** Limitations in existing work of mechanically thrown objects tracking.

Ref.	Year	Trajectory Type	Prediction Algorithm	Results (Accuracy)	Limitation(s)
[22]	2020	Mechanical ball throws using ping-pong playing robot. Observe its flight through 3 cameras (right, left and auxiliary) of speed 169 FPS	Dual Neural Network	300 trajectories for the training set and 30 trajectories for the test set. The test results in absolute mean error of 36.6 mm and standard deviation of 18.8 mm	Limited training and testing
[23]	2020	Mechanical ball throw using ping-pong playing robot and observe its flight (0.8 to 1.2 s) through 4 RGB cameras of speed 180 FPS (Frames Per Second) attached at ceiling	Variational auto-encoder deep NN	614 trajectories for the training set (90% training and 10% for validation) and 35 trajectories for the test set. Prediction’s absolute mean error converges to nearly 40–60 mm based upon observations in first 40–50 frames of flight trajectory.	Error is high but could be improved with more training trajectories
[24]	2019				
[25]	2020				
[29]	2010	Ball throws using mechanical device. Observe its actual positions in flight with the help of photoelectric sensors. Flight is also captured by single camera of 87 FPS speed.	Observations of ball positions through photoelectric sensors and Size based Tracking of ball through 2D coordinates in image plane are further passed to EKF for prediction of final 2D impact point on DST-Touch screen	The accuracy was measured in 17 test throws only and for final 2D impact position (on a DST-Touch kit) only. The average error deviation of final impact position was 1.20 mm to 3.98 mm.	(1) It is assumed that the line of sight is perpendicular to the camera’s measuring plane (2) Photoelectric sensors were used to get actual interception position of ball whereas in practical industrial scenarios such sensors are not easily implementable
[30]	2009				
[31]	2008				
[32]	2016	Ball throws using mechanical device. Observe its flight trajectory through stereo vision of 2 cameras (left and right) of spatial resolution 2048 × 2048 and speed was not specified in their articles.	kNN (k-Nearest Neighbor) Regression	2048 real-world trajectories were saved in the database and then testing were performed on 150 trajectories. First 40 frames ball positions were used during testing and after applying KNN the prediction was within 30 mm for 92% of trajectories.	Error is high but could be improved with: (1) More training and better pattern recognizer (such as deep neural networks) (2) Increasing the number of observations (i.e., frames) for prediction
[33]	2015				
[34]	2013	The mechanical throws were simulated using physical motion model. Each sample trajectory was obtained in nearly 2.5 m long flight of tennis ball.	Neural Network with one hidden layer was used to train 15 simulated trajectory sets whereas each set had 10 sample trajectories	The mean error was nearly 24 to 26 mm between measured values and prediction results in simulated environment.	Being results in simulated environment, this error is high. Also very limited training and testing
[35]	2017	Ball throws using mechanical device. Observe its flight trajectory through stereo vision of 2 cameras (left and right) of spatial resolution 2048 × 2048 and speed was not specified in their articles.	Deterministic motion model further governed by genetic programming algorithm	Their algorithm was tested through MSE (Mean Square Error) in chosen frames 60 to 80 only and in only 20 test trajectories. The average mean square error (MSE) in 20 trajectories was 5.4 mm	Average MSE was good but it was based upon just 20 testing trajectories as well as the error was calculated within selected frames (60 to 80) and it does not reflect the error of whole flight trajectory
[36]	2018				
[37]	2019				
[38]	2017				

**Table 2:** Final touch point accuracy by different multi-camera setups (results based upon average of 50 real world experiments).

Multicamera Setups	Final 3D Interception Position Error (Average Error—in mm)		
	*X*-Axis	*Y*-Axis	*Z*-Axis
C2 + C4	2.5	1.7	5.4
C1 + C4	1.9	5.2	2.0
C2 + C3	2.5	6.5	3.1
C1 + C3	2.8	4.5	1.5
C1 + C2 + C4	1.5	5.0	1.9
C1 + C2 + C3	2.3	4.5	1.7
C2 + C3 + C4	1.1	2.0	1.4
**C1 + C3 + C4**	**0.9**	**1.8**	**1.6**

## Data Availability

Our dataset is not publicly available yet. However, in future, we will make it publically available along with code on GitHub.
